# Editorial: Advanced chemical and material tools to enhance the therapeutic effect of drugs

**DOI:** 10.3389/fphar.2023.1239882

**Published:** 2023-08-07

**Authors:** Ayesha Ihsan, Mubashar Rehman

**Affiliations:** ^1^ National Institute for Biotechnology and Genetic Engineering College, Pakistan Institute of Engineering and Applied Sciences (NIBGE-C, PIEAS), Faisalabad, Pakistan; ^2^ Department of Pharmacy, Quid-i-Azam University, Islamabad, Pakistan

**Keywords:** chemical tools, material tools, drug delviery, drug crystals, multidrug resistance, targeting of durg receptors

In the world of functional medicine and therapy, the research of the last 10 years manifested the advanced chemical and material tools as the game-changers of the field. Different innovations in this field offer unprecedented opportunities to strengthen the frontiers of clinical decision making and even tackling unexpected disease outbreaks. In parallel to the exponential growth of genetic databases with smart data processing tools, advanced chemical and material synthesis techniques have been designed as innovative therapeutic solution or as investigational diagnostic agent. In this scenario, scientist become enabled to study even more diverse mechanisms of various drug targets, pharmacological action of drugs and their molecular origin. All this progress is shaping the concept of precision medicine into reality. To this end, precisely designed chemical tools and materials are perpetually required for more specific and effective prevention and treatment modalities. Hence, this Research Topic is focused to spotlight the latest chemical and material tools to improve treatment, diagnosis and bench scale monitoring technique.

This research theme collected different contributions and highlights on multiple unique approaches on the functional upgradation of drug molecules, drug targets, their selected delivery and sensitive detection to deal more prevalent health problems and their diagnosis. These contributions enabled us to shed light on the diversity and progression of advanced chemical and material tools in this field.

Among different health threats, drug-resistant pathogens pose a significant menace to public health worldwide. The emergence and spread of antibiotic-resistant pathogens have led to increased mortality rates, prolonged illness, and higher healthcare costs. Screening or identifying new potential drug candidates, can combat the resistant pathogens and provide effective treatment options. In this Research Topic, Javaid et al. screened and characterized a new traizole Schiff base moieties while considering its pharmacological compatibilities, antimicrobial and cytotoxic potential. Besides, the discovery of new drugs, understanding and characterizing the existent drug crystal polymorphs can provide valuable insights on the structure property relationship for improving both preventive and therapeutic medicines. Researchers and companies continue to explore the occurrence, characterization, and impact of polymorphs to optimize drug development and ensure the production of safe and effective medications. In this Research Topic, Gorin et al. studies different polymorphic form of laterpridine and their pharmacological and cognitive enhancing neurotropic activities.

Regulation of specific drug receptors in human body have been acknowledged as an effective therapy for treating different neurological and inflammatory disorders. Till date, numerous receptor inhibitors have been screened and identified. However, achieving target selectivity is still a big unmet challenge. In this Research Topic, Chang et al. highlighted specific gel-forming antagonists which may represent promising candidates for sustained and specific inhibition of CGRP targets. These gel-forming antagonists could also be used to deliver medication directly to the site of action and may provide localized relief.

Despite of looking into the frontiers of target specific efficient drug and their bioavailability, sensitive bench monitoring analysis plays a crucial role to quantify the clinical outcome of therapeutic interventions and pharmacokinetic variation in patients. Developing an Ultra-Performance Liquid Chromatography (UPLC) method for drug detection in plasma is of significant importance in various areas, such as pharmaceutical research, clinical diagnostics, forensic toxicology, and drug development. The studies of Harahap et al. for development and validation of a micro sampling technique for drug quantification, is included in this Research Topic.

Based on these achievements, we anticipate that better chemical tools and advanced materials can be rationalized for improving the safety and efficiency of various diagnostics and therapeutics modalities.

